# Exploring the key ferroptosis-related gene in the peripheral blood of patients with Alzheimer’s disease and its clinical significance

**DOI:** 10.3389/fnagi.2022.970796

**Published:** 2022-09-01

**Authors:** Xiaonan Wang, Yaotian Tian, Chunmei Li, Min Chen

**Affiliations:** ^1^Department of Radiology, Beijing Hospital, National Center of Gerontology, Institute of Geriatric Medicine, Chinese Academy of Medical Sciences, Beijing, China; ^2^Graduate School of Peking Union Medical College, Chinese Academy of Medical Sciences, Beijing, China

**Keywords:** Alzheimer’s disease, ferroptosis, GEO, diagnosis, immune infiltration

## Abstract

**Introduction:**

Alzheimer’s disease (AD) is the most common type of dementia, and there is growing evidence suggesting that ferroptosis is involved in its pathogenesis. In this study, we aimed to investigate the key ferroptosis-related genes in AD and identify a novel ferroptosis-related gene diagnosis model for patients with AD.

**Materials and methods:**

We extracted the human blood and hippocampus gene expression data of five datasets (GSE63060, GSE63061, GSE97760, GSE48350, and GSE5281) in the Gene Expression Omnibus database as well as the ferroptosis-related genes from FerrDb. Differentially expressed ferroptosis-related genes were screened by random forest classifier, and were further used to construct a diagnostic model of AD using an artificial neural network. The patterns of immune infiltration in the peripheral immune system of AD were also investigated using the CIBERSORT algorithm.

**Results:**

We first screened and identified 12 ferroptosis-related genes (*ATG3, BNIP3, DDIT3, FH, GABARAPL1, MAPK14, SOCS1, SP1, STAT3, TNFAIP3, UBC*, and *ULK*) via a random forest classifier, which was differentially expressed between the AD and normal control groups. Based on the 12 hub genes, we successfully constructed a satisfactory diagnostic model for differentiating AD patients from normal controls using an artificial neural network and validated its diagnostic efficacy in several external datasets. Further, the key ferroptosis-related genes were found to be strongly correlated to immune cells infiltration in AD.

**Conclusion:**

We successfully identified 12 ferroptosis-related genes and established a novel diagnostic model of significant predictive value for AD. These results may help understand the role of ferroptosis in AD pathogenesis and provide promising therapeutic strategies for patients with AD.

## Introduction

Dementia is a group of symptoms impacting memory, thinking, and behavior, affecting more than 55 million people worldwide; Alzheimer’s disease (AD) is the most common and well-known type of dementia, accounting for 60–80% of all cases, resulting in a considerable burden to society (Alzheimer’s Disease International, 2021). AD is characterized pathologically by amyloid-β (Aβ) plaques aggregation and tau neurofibrillary tangles accumulation. Recent studies have also indicated the crucial roles of oxidative stress (Butterfield and Boyd-Kimball, 2018), autophagy (Uddin et al., 2018), and neuroinflammation (Heneka et al., 2015) in the pathological mechanism of AD. However, despite decades of research, the precise mechanism of AD remains uncertain.

Meanwhile, the diagnosis and effective treatments for AD are challenging. The hippocampus, which is the center of memory, thinking and learning in the brain, is thought to be closely associated with the development of AD (Lazarov and Hollands, 2016). Although many imaging methods are in great interest for hippocampal analysis, the accurate diagnosis rely on biopsy, which is difficult to perform in the clinic. Although several novel biomarkers in cerebrospinal fluid, including amyloid-b (Aβ42), total tau, and phosphorylated tau, have been recommended for the diagnosis of AD, reflecting favorable diagnostic accuracy (Olsson et al., 2016); they were limited to the large-scale clinical screening application due to the invasive collection process and high cost. Thus, determining peripheral blood-based biomarkers, which is non-invasive and cost-effective, has become a promising research direction in AD (Olsson et al., 2016; Tang and Liu, 2019). Notably, previous studies have highlighted that multiple genes could also concerned within the pathogenesis and various biological activities of AD. Trying to find common genes differentially expressed in the peripheral blood cells and brain tissue is one of the most interesting aspects of investigating AD, while several studies have stressed that the expression of some genes from the peripheral blood might be related to the pathological change in AD patients’ brain tissue (Yu et al., 2021; Wang et al., 2022). Therefore, a comprehensive investigation of the transcriptomics characteristics and establishment of a diagnostic model based on the peripheral blood may help understand the underlying pathogenesis and diagnosis of AD.

Ferroptosis is a newly discovered type of regulated cell death characterized by iron-dependent lipid peroxidation; it has attracted growing attention in recent years (Dixon et al., 2012). Numerous studies have illustrated that ferroptosis plays a pivotal role in the mechanisms of cancer, tumor immunity, and ischemic disease. However, few studies focus on its role in neurodegenerative diseases, especially in AD (Yan et al., 2021). Recently, the evidence of iron elevation and lipid peroxidation products in the AD brain implicates the role of ferroptosis in the pathogenesis of AD. Growing proof suggests that ferroptosis potentially participates within the pathogenesis of AD and may promise a promising therapeutic target for AD (Chen et al., 2021; Zhang et al., 2021). While several prior reviews have mainly focused on discussing how ferroptosis participates in the AD (Jakaria et al., 2021; Majerníková et al., 2021) and investigating whether ferroptosis as a drug target of AD is effective in delaying the progression of AD (Vitalakumar et al., 2021). To our knowledge, there have been no studies reporting the relationship between ferroptosis genes in peripheral blood and AD. In this study, we aimed to conduct a bioinformatics analysis using human blood gene expression data from the Gene Expression Omnibus database to identify the differential ferroptosis-related genes in AD and build a ferroptosis-related gene diagnosis model of AD.

## Materials and methods

### Data sources

**FIGURE 1:**
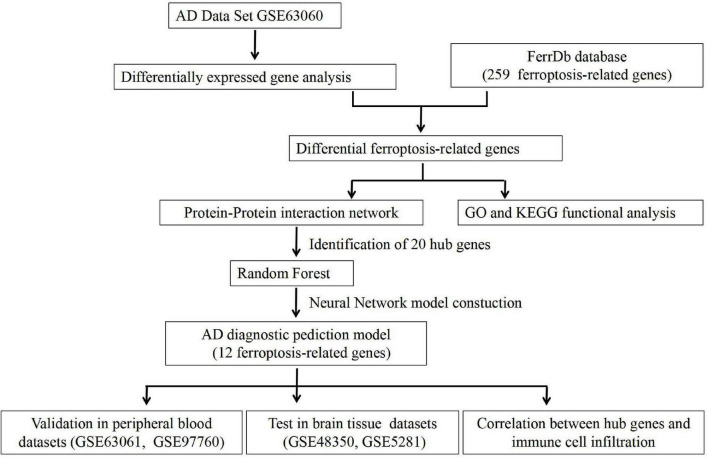
The flow chart of this study.

Figure 1 shows the flow chart of our study. The mRNA expression files of patients with AD were downloaded from five datasets in the Gene Expression Omnibus database, including three peripheral blood datasets (GSE63060, GSE63061, and GSE97760) and two hippocampus datasets (GSE48350 and GSE5281) (Berchtold et al., 2013; Naughton et al., 2015; Sood et al., 2015; Readhead et al., 2018). Detailed info concerning these datasets is summarized in Table 1. Meanwhile, 259 ferroptosis-related genes were extracted and analyzed from the Ferroptosis database (Supplementary Table 1).^1^

**TABLE 1:** Detailed information of the included datasets.

Dataset	Platform	Normal control sample	AD sample	Resource
GSE63060	GPL6947 (Illumina HumanHT-12 V3.0 expression beadchip)	104	145	Peripheral blood
GSE63061	GPL10558 (Illumina HumanHT-12 V4.0 expression beadchip)	134	139	Peripheral blood
GSE97760	GPL16699 [Agilent-039494 SurePrint G3 Human GE v2 8 × 60 K Microarray 039381 (Feature Number version)]	9	10	Peripheral blood
GSE48350	GPL570 [(HG-U133_Plus_2) Affymetrix Human Genome U133 Plus 2.0 Array]	42	19	Hippocampus
GSE5281	GPL570 [(HG-U133_Plus_2) Affymetrix Human Genome U133 Plus 2.0 Array]	13	10	Hippocampus

AD, Alzheimer’s disease.

### Identification of candidate ferroptosis-related genes

Differentially expressed genes (DEGs) between normal control and AD samples in GSE63060 were determined using the “limma” R package (Ritchie et al., 2015) (adjusted *p* value < 0.05) and visualized using heatmaps. Then we intersected these DEGs with ferroptosis-related genes to identify candidate ferroptosis-related genes.

### Analysis of differential ferroptosis-related genes

The “Metascape” website (Zhou et al., 2019), Gene Ontology and Kyoto Encyclopedia of Genes and Genomes analysis by the “clusterProfiler” R package (Yu et al., 2012) were used for the functional enrichment analyses of differential ferroptosis-related genes. Additionally, the STRING database^2^ (Szklarczyk et al., 2015) was used to establish a protein-protein interaction (PPI) network of the ferroptosis DEGs.

### Development of the random forest and neural network model

We first used the GSE63060 dataset as the training cohort to construct a random forest model via the randomForest R package (Alderden et al., 2018). The genes with an importance value greater than 2 and ranked in the top 12 were chosen as the disease specific genes for the subsequent model construction. Then, we used the neuralnet R package (Beck, 2018) for constructing an artificial neural network model of the pivotal ferroptosis-related genes. Five hidden layers were set as the model parameters to construct a classification model of AD through the obtained gene weight information. The classification score of the obtained disease neural network model was calculated using the following formula: neuroAD = ∑GeneExpression*NeuralNetworkWeight. The receiver operating characteristic (ROC) curves with area under curve (AUC) values were used to estimate the predicted performance of the model to classify the AD and the normal samples. The classification efficacy was then external validated using four independent datasets: GSE63061, GSE97760, GSE48350, and GSE5281.

### Exploration of immune cell infiltration

The CIBERSORT algorithm (Chen et al., 2018) was carried out to quantify the relative abundance of 22 types of infiltrating immune cells in the AD and normal-aging samples, and *p* < 0.05 was regarded as statistically significant. Correlations between the targeted ferroptosis-related genes and immune cells were evaluated using the Wilcoxon rank-sum test and Spearman correlation analysis.

### Statistical analysis

All analyses were performed using R v.4.0.3.^3^ The Wilcoxon rank-sum test was used to compare two groups, and the correlations were determined using Spearman correlation analysis. Statistical significance was set at a two-tailed *p*-value < 0.05.

## Results

### Detection of ferroptosis differentially expressed genes

**FIGURE 2:**
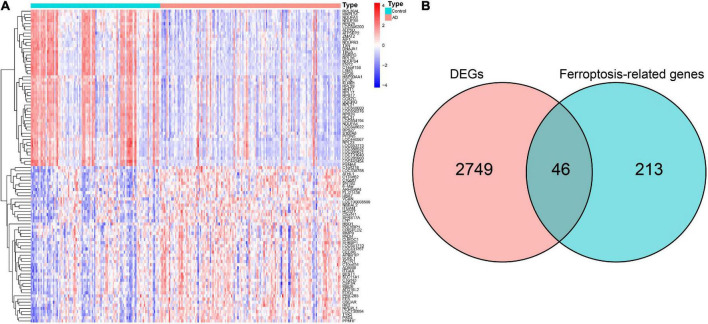
Identification of hub ferroptosis-related genes. **(A)** Heatmap for the top 50 upregulated and downregulated DEGs. **(B)** Venn diagram showing candidate ferroptosis-related genes. DEGs, differentially expressed genes.

The GSE63060 dataset contained 104 normal control and 145 AD peripheral blood samples. Notably, 2,795 DEGs between the normal control and AD samples were first screened (Supplementary Table 2), and the high fifty upregulated and downregulated DEGs are listed in Figure 2A. By intersecting these DEGs and 259 ferroptosis-related genes, 46 overlapping ferroptosis DEGs (34 upregulated and 12 downregulated) were further obtained (Figure 2B and Supplementary Table 3).

### Functional enrichment analysis

**FIGURE 3:**
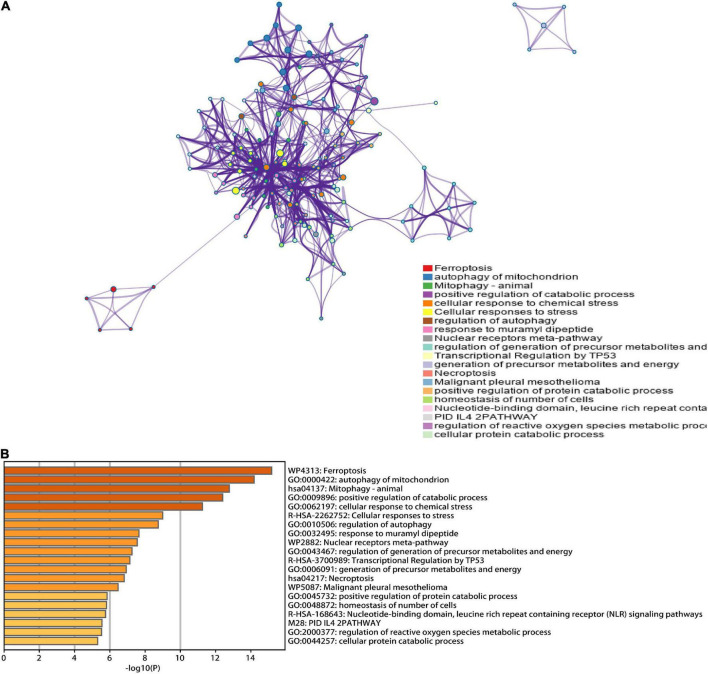
Functional analysis of 46 hub ferroptosis-related genes. **(A)** Network of enriched terms. **(B)** Bar graph of biological pathways according to the *p*-value.

**FIGURE 4:**
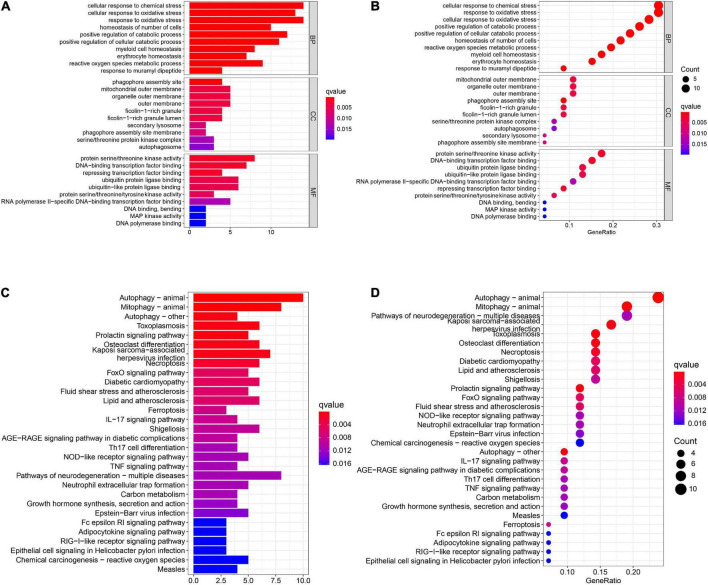
The GO and KEGG analysis. The **(A)** bar plot and **(B)** bubble of GO enrichment analysis. The **(C)** bar plot and **(D)** bubble of KEGG enrichment analysis.

A total of 46 DEGs were first uploaded to the Metascape online tool to investigate the potential biological process and pathway. We found that these genes were remarkably enriched in Ferroptosis, autophagy of mitochondrion, positive regulation of catabolic processes, regulation of autophagy, regulation of generation of precursor metabolites and energy, necroptosis, positive regulation of protein catabolic process, and regulation of reactive oxygen species metabolic process (Figure 3). Furthermore, Gene Ontology (GO) analysis and Kyoto Encyclopedia of Genes and Genomes (KEGG) pathway analysis similarly revealed that these ferroptosis-related genes were significantly enriched in ferroptosis, autophagy, response to oxidative stress, TNF signaling pathway, and IL-17 signaling pathway (Figure 4).

### Construction and external validation of a ferroptosis-related gene diagnostic model

**FIGURE 5:**
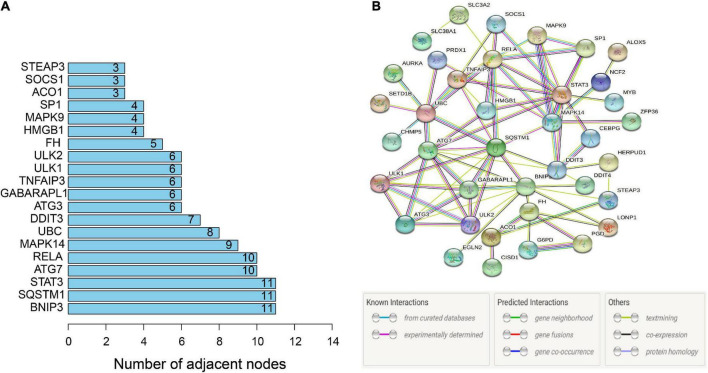
PPI network. **(A)** PPI network of 46 ferroptosis-related genes. **(B)** Bar graph identifying the top 20 hub genes, ranked based on the number of nodes with which they are connected. PPI, protein-protein interaction.

**FIGURE 6:**
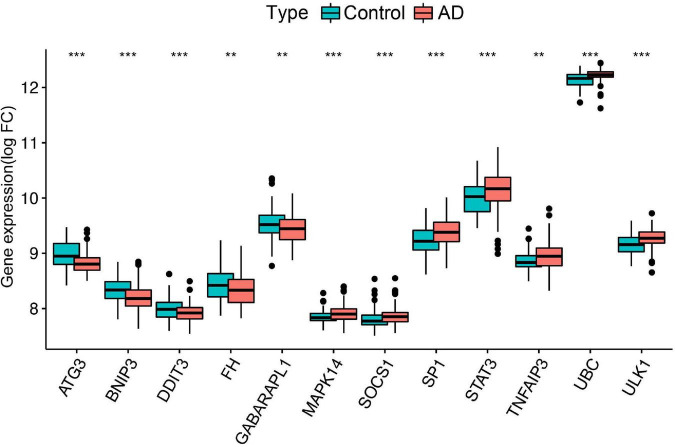
Differential expression of the 12 ferroptosis-related genes between normal controls and AD, **p < 0.01; ***p < 0.001. FC, fold changes.

**FIGURE 7:**
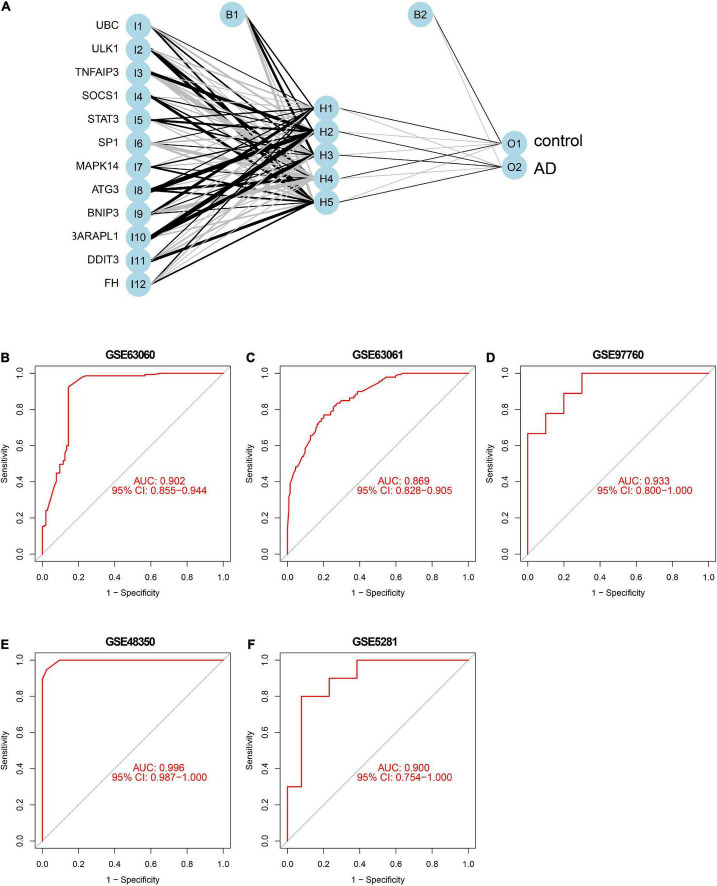
Neural network and ROC curve analysis. **(A)** Neural network visualization curves of **(B)** GSE63060, **(C)** GSE63061, **(D)** GSE97760, **(E)** GSE48350, and **(F)** GSE5281, respectively. AUC, area under the curve.

To further identify the candidate hub genes among these differential ferroptosis-related genes, the PPI interaction network was constructed (Figure 5A). Figure 5B depicts the top 20 hub genes, ranked according to their connectedness in the PPI network. Next, we input the 20 DEGs into the random forest classifier to further identify 12 critical genes, including *ATG3, BNIP3, DDIT3, FH, GABARAPL1, MAPK14, SOCS1, SP1, STAT3, TNFAIP3, UBC*, and *ULK1*. The relative expression levels of the 12 hub genes differed significantly between AD and normal control based on GSE63060 data (Figure 6). Subsequently, we constructed an artificial neural network model for classifying the normal control and AD samples based on the 12 genes, with the settings of 12 input layers, five hidden layers, and two output layers (Figure 7A). The receiver operating characteristic curves showed that the model accurately classified AD samples and normal controls in peripheral blood tissues, and the AUC was 0.902 (Figure 7B). The detailed classification of each sample as predicted by the model is presented in Supplementary Table 4. Additionally, to addition assess whether or not this model is worth using in clinical practice, we externally tested our model on four independent datasets. In the two peripheral blood datasets, GSE63061 (normal controls = 134, AD = 139), GSE97760 (normal controls = 9, AD = 10), the AUC was 0.869 and 0.933, respectively (Figures 7C,D). Simultaneously, Figures 7E,F show the receiver operating characteristic curves verified by two hippocampus datasets, GSE48350 (normal controls = 42, AD = 19) and GSE5281 (normal controls = 13, AD = 10). The AUC were 0.996 and 0.900, respectively. These results indicate that our model classification performance was robust and stable.

### Analysis of immune cell infiltration

**FIGURE 8:**
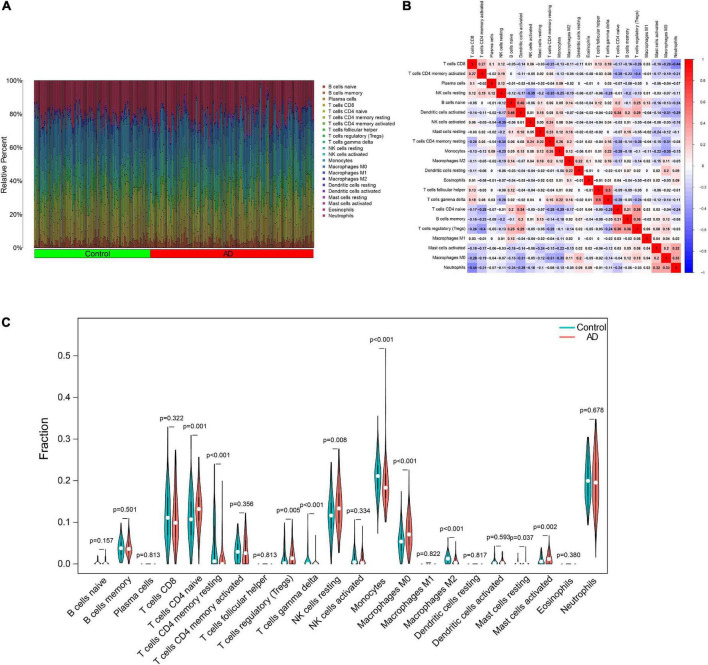
Characteristics of immune cell infiltration. **(A)** Proportions of 22 types of immune cells. **(B)** Heatmap of the correlation among 22 types of immune cells. **(C)** Differential immune cell infiltration between AD and control groups. AD, Alzheimer’s disease.

In particular, we comprehensively investigated the immune cell infiltration characteristics in the peripheral blood of normal control and AD patients. Figures 8A,B display the 22 distinct infiltrated immune cells. Besides, we found that the fractions of 10 types of immune cells differed significantly between normal and AD samples. Among them, naive CD4 T cells, regulatory T cells (Tregs), resting NK cells, M0 macrophages, and activated mast cells were highly infiltrated in the AD group. In contrast, monocytes and M2 macrophages were significantly lower in the patients in the AD group. However, resting memory CD4 T cells, resting mast cells, and gamma delta T cells were finally excluded due to the significantly lower proportions in all samples (Figure 8C). By combining difference and correlation analyses, we found that several types of immune cells, especially CD4 T cells, monocytes, and NK cells were strikingly associated with almost all the 12 ferroptosis-related genes (Supplementary Figures 1, 2 and Supplementary Table 5). These findings suggested that these ferroptosis-related genes may have immunomodulatory roles in the AD.

## Discussion

Accumulating evidence suggests that ferroptosis, a nonapoptotic, iron-dependent, and lipid peroxidation-driven type of programmed cell death, plays a important role within the pathologic process of AD (Sood et al., 2015; Chen et al., 2021; Zhang et al., 2021). Researchers have expended significant effort to elucidate the possible mechanism of ferroptosis involved in the pathological process of AD and to exploit its promising potential clinical application in AD (Majerníková et al., 2021). However, the relationship between ferroptosis-related genes and AD remains unclear. This study systematically screened and determined 12 ferroptosis-related genes, comprehensively explored the association between ferroptosis-related genes and AD, and constructed a reliable diagnostic model for AD.

Several pivotal biological pathways of ferroptosis have been reported in AD pathology, such as iron dyshomeostasis, oxidative stress and lipid peroxidation, and the reduced glutathione (GSH) and glutathione peroxidase (*GPX4*) levels (Ashraf et al., 2020). Some revealed differentially expressed ferroptosis-related genes in AD can affect mostly neurons, contributing to tau phosphorylation and Aβ accumulation, such as acyl-CoA synthetase long-chain family member (*ACSL4*) and *GPX4*, which were tightly related to lipid peroxidation of ferroptosis (Ashraf et al., 2020; Kim et al., 2021). In our study, the differentially expressed ferroptosis-related genes were primarily associated with the response to oxidative stress, indicating one of the possible mechanisms of these genes involved in AD. Meanwhile, we noticed that the autophagy pathway was also actively enriched in the functional analysis, uncovering the close connection between ferroptosis and autophagy, which was also pointed out in a previous study (Zhou et al., 2020). Besides, studies supported that the TNF signaling pathway and IL-17 signaling pathway can also play crucial roles in the ferroptosis in AD, consistent with our findings (Fischer and Maier, 2015; Milovanovic et al., 2020).

Among the 12 ferroptosis-related genes included in the diagnostic model, autophagy-related genes 3 (*ATG3*) is one of the key genes involved in autophagy, which can also contribute to ferroptotic cell death (Zhou et al., 2020). The expression and modification of *ATG3* play important roles in erastin-induced ferritin degradation, iron accumulation and lipid peroxidation, as well as subsequent ferroptosis (Hou et al., 2016). Bcl-2/adenovirus E1B 19-kDa interacting protein (*BNIP3*) is a death inducing mitochondrial protein that is a member of the Bcl-2 family without a functional BH3 domain, has been suggested affect Aβ-induced neuronal death in AD (Zhang et al., 2007). Researchers have noticed that *GABARAPL1* can confer an affinity for several mutated proteins that form aggregates in neurodegenerative diseases, and the degradation of *GABARAPL1* can induce these neurodegenerative diseases to progress, further suggesting the importance of *GABARAPL1* in the prevention of neurodegenerative diseases (Simunovic et al., 2009; Le Grand et al., 2011). Mitogen-activated protein kinase 14 (*MAPK14*), a modulator of the innate immune system, has been found significantly upregulated in a mouse model of AD, leading to the increased BACE1 levels and plaque formation (Alam and Scheper, 2016). The activation of the MAPK signaling pathway was also indicated to be involved in ferroptosis in the AD pathological process (Kheiri et al., 2018). The *MAPK14* expression can be inhibited by miR-22-3p overexpression, thereby reducing of Aβ deposit and alleviating AD symptoms (Ji et al., 2019). Silencing of cytokine signaling factor 1 (*SOCS1*) plays a significant role in immune reaction by modulating several cytokines, inducing the neuroinflammation alteration in AD (Guo et al., 2021). Citron et al. reported that specificity protein 1 (*SP1*) was overexpressed in the brains of human and transgenic AD model mice, which can positively modulate the expression levels of several AD-related proteins, including amyloid precursor protein and tau. Therefore, it can serve as a potential therapeutic target (Citron et al., 2008, 2015). Signal transducer and activator of transcription 3 (*STAT3*) was demonstrated to be associated with BACE1 levels and neuroinflammation in AD (Reichenbach et al., 2019). Inhibition of *STAT3* can reduce LPS-induced microglial activation and the levels of cytokines IL-6, IL-1β, and TNF-α in the AD hippocampus (Millot et al., 2020). TNF alpha-induced protein 3 (*TNFAIP3*) is an anti-inflammatory factor that can improve cell survival by inhibiting the expression of inflammatory NF-κB signaling (Catrysse et al., 2014). Previous studies have reported that *TNFAIP3* overexpression can promote oxygen-free radical generation and ferroptosis and may relate to neurodegenerative diseases, such as multiple sclerosis and Parkinson’s disease (Perga et al., 2017; Xiao et al., 2019). Ubiquitin C (*UBC*) is an essential source of ubiquitin in the process of cell proliferation and stress. It has been found that the ubiquitin proteasome system (UPS) play a crucial role in the pathogenesis of AD, as the existence of Ub immunoreactivity in AD-linked neuronal inclusions, including neurofibrillary tangles, is observed in all types of AD cases, and targeted treatment at the main components of these pathways has a great perspective in advancing new therapeutic interventions for AD (Al Mamun et al., 2020). All these findings probably offer promising research direction for AD in the future. Although the remaining three ferroptosis-related genes had been primarily reported to be involved in cancer, their detail role in AD is unclear. Thus, further research is warranted to explore it.

It is currently believed that neuroinflammation is responsible for the pathogenic process in AD, and the peripheral immune cells similarly participated in the course (Heneka et al., 2015). Strong inflammatory reactions mediated by resident brain cells and peripheral immune cells, which infiltrate the brain at various stages of disease progression, were found in AD patients’ brain. As the immune and inflammatory environment in the peripheral blood change, the metabolism, signaling, and biological process in brain tissue also change (Olsen and Singhrao, 2016). Therefore, we thoroughly analyzed the infiltration of immune cells in AD. In the present study, we noticed that the abnormal distribution of the peripheral immune cells in AD. Among these immune cells, CD4 T cells were more infiltrated in AD samples than controls, similar to a recent study (Xu and Jia, 2021). Lueg et al. (2015) revealed that an increased proportion of CD4 T cells in peripheral blood is positively correlated with cognitive defects and magnetic resonance imaging changes of specific brain regions in AD patients. Sutphen et al. (2015) reported that severe inflammatory reactions in middle-aged people can increase the risk of AD. These findings suggest the disturbance of immune infiltration in the peripheral immune system plays a significant role in AD. Although immune abnormalities do not necessarily lead to AD, we speculate that elderly people with long-term immune system abnormalities may have a much higher risk of AD than the normal elderly population. Thus, the treatment or intervention of early inflammatory reactions in elderly people may help delay the onset and development of AD. However, our current study did not determine abnormal cut-off values of the immune cells, and large-sample data comparing normal-aging individuals, and patients with mild cognitive impairment or AD are necessary for future studies. In particular, previous studies also suggest that the involvement of ferroptosis in the pathological process of neuroinflammation in AD (Cheng et al., 2021). Here, we comprehensively detected the association between the 12 ferroptosis-related genes and peripheral immune cells in AD. Infiltration levels of CD4 T cells, monocytes, and NK cells notably differed between the differential expression groups of nearly all the 12 ferroptosis-related genes, and were significantly correlated with the 12 ferroptosis-related genes expression. Thus, more studies should be conducted in the future to exploit the underlying mechanisms of ferroptosis as well as the link between ferroptosis and inflammation in AD, which can possibly identify new targets for treatment.

Growing evidence indicated that ferroptosis is a potential therapeutic target for AD (Uddin et al., 2018; Chen et al., 2021). CMC121 was found to alleviate cognitive loss by modulating lipid metabolism and reducing inflammation and lipid peroxidation via inhibiting the fatty acid synthase *in vitro* and *in vivo* models of AD (Ates et al., 2020). Chalcones 14a-c was demonstrated as a potential candidate for AD treatment via simultaneous inhibition of Aβ and lipid peroxidation (Cong et al., 2019). Additionally, previous studies have indicated that N-acetylcysteine (NAC) can prevent the spatial memory impairments by reducing lipid oxidation and inducing hippocampal total GSH levels in AD animal models, and the improvement of behavioral symptoms of AD patients after treatment with NAC was also found in the clinical trial (Adair et al., 2001; More et al., 2018). These findings suggest that ferroptosis inhibition could provide a new promising therapeutic strategy for AD, and large, randomized clinical trials of anti-ferroptotic drugs in the treatment of AD are warranted.

This study has some limitations. First, our ferroptosis-related gene diagnostic model was established and validated by retrospective public data from the Gene Expression Omnibus database; further independent and prospective cohorts are required to verify this model. Also, although we enrolled generally approved ferroptosis-related genes in the FerrDb, it is necessary to incorporate more newly identified ferroptosis-related genes. In addition, given the lack of detailed information, some important clinical characteristics, such as age and gender, were not integrated into the diagnostic model. Finally, further experimental validations are needed to identify the detailed molecular mechanism underlining these ferroptosis-related genes in AD.

In conclusion, we identified 12 crucial ferroptosis-related genes and developed a novel diagnostic model for AD, which showed significant predictive performance. This study may be useful in understanding the molecular mechanisms of ferroptosis involved in AD pathogenesis and in investigating the optimal therapeutic strategies for patients with AD.

## Data availability statement

The original contributions presented in this study are included in the article/Supplementary material, further inquiries can be directed to the corresponding author.

## Author contributions

XW and MC designed the work. XW collected and integrated the data. XW, YT, and CL analyzed the data and prepared the manuscript. CL and MC edited and revised the manuscript. All authors read and approved the final manuscript.

## Footnotes

1
http://zhounan.org/ferrdb

2
http://string-db.org/

3
http://www.R-project.org

## Abbreviations

AD: Alzheimer’s disease
ROC: receiver operating characteristic
AUC: area under curve
DEGs: differentially expressed genes
PPI: protein-protein interaction.

## References

[B1] Adair J. C. Knoefel J. E. Morgan N. (2001). Controlled trial of N-acetylcysteine for patients with probable Alzheimer’s disease. *Neurology* 57 1515–1517. 10.1212/WNL.57.8.1515 11673605

[B2] Al Mamun A. Rahman M. M. Zaman S. Munira M. S. Uddin M. S. Rauf A. (2020). Molecular insight into the crosstalk of UPS components and Alzheimer’s disease. *Curr. Protein Pept. Sci.* 21 1193–1201. 10.2174/1389203721666200923153406 32964822

[B3] Alam J. Scheper W. (2016). Targeting neuronal MAPK14/p38α activity to modulate autophagy in the Alzheimer disease brain. *Autophagy* 12 2516–2520. 10.1080/15548627.2016.1238555 27715387 PMC5173254

[B4] Alderden J. Pepper G. A. Wilson A. Whitney J. D. Richardson S. Butcher R. (2018). Predicting pressure injury in critical care patients: A machine-learning model. *Am. J. Crit. Care.* 27 461–468. 10.4037/ajcc2018525 30385537 PMC6247790

[B5] Alzheimer’s Disease International (2021). *World Alzheimer Report 2021: Journey through the diagnosis of dementia.* London: Alzheimer’s Disease International.

[B6] Ashraf A. Jeandriens J. Parkes H. G. So P. W. (2020). Iron dyshomeostasis, lipid peroxidation and perturbed expression of cystine/glutamate antiporter in Alzheimer’s disease: Evidence of ferroptosis. *Redox Biol.* 32:101494. 10.1016/j.redox.2020.101494 32199332 PMC7083890

[B7] Ates G. Goldberg J. Currais A. Maher P. (2020). CMS121, a fatty acid synthase inhibitor, protects against excess lipid peroxidation and inflammation and alleviates cognitive loss in a transgenic mouse model of Alzheimer’s disease. *Redox Biol.* 36:101648. 10.1016/j.redox.2020.101648 32863221 PMC7394765

[B8] Beck M. W. (2018). NeuralNetTools: Visualization and analysis tools for neural networks. *J. Stat. Softw.* 85 1–20. 10.18637/jss.v085.i11 30505247 PMC6262849

[B9] Berchtold N. C. Coleman P. D. Cribbs D. H. Rogers J. Gillen D. L. Cotman C. W. (2013). Synaptic genes are extensively downregulated across multiple brain regions in normal human aging and Alzheimer’s disease. *Neurobiol. Aging* 34 1653–1661. 10.1016/j.neurobiolaging.2012.11.024 23273601 PMC4022280

[B10] Butterfield D. A. Boyd-Kimball D. (2018). Oxidative stress, amyloid-β Peptide, and altered key molecular pathways in the pathogenesis and progression of Alzheimer’s disease. *J. Alzheimers Dis.* 62 1345–1367. 10.3233/JAD-170543 29562527 PMC5870019

[B11] Catrysse L. Vereecke L. Beyaert R. van Loo G. (2014). A20 in inflammation and autoimmunity. *Trends Immunol.* 35 22–31. 10.1016/j.it.2013.10.005 24246475

[B12] Chen B. Khodadoust M. S. Liu C. L. Newman A. M. Alizadeh A. A. (2018). Profiling tumor infiltrating immune cells with CIBERSORT. *Methods Mol. Biol.* 1711 243–259. 10.1007/978-1-4939-7493-1_12 29344893 PMC5895181

[B13] Chen K. Jiang X. Wu M. Cao X. Bao W. Zhu L. Q. (2021). Ferroptosis, a potential therapeutic target in Alzheimer’s disease. *Front. Cell Dev. Biol.* 9:704298. 10.3389/fcell.2021.704298 34422824 PMC8374166

[B14] Cheng Y. Song Y. Chen H. Li Q. Gao Y. Lu G. (2021). Ferroptosis mediated by lipid reactive oxygen species: A possible causal link of neuroinflammation to neurological disorders. *Oxid. Med. Cell. Longev.* 2021:5005136. 10.1155/2021/5005136 34725564 PMC8557075

[B15] Citron B. A. Dennis J. S. Zeitlin R. S. Echeverria V. (2008). Transcription factor Sp1 dysregulation in Alzheimer’s disease. *J. Neurosci. Res.* 86 2499–2504. 10.1002/jnr.21695 18449948

[B16] Citron B. A. Saykally J. N. Cao C. Dennis J. S. Runfeldt M. Arendash G. W. (2015). Transcription factor Sp1 inhibition, memory, and cytokines in a mouse model of Alzheimer’s disease. *Am. J. Neurodegener. Dis.* 4 40–48. 26807343 PMC4700125

[B17] Cong L. Dong X. Wang Y. Deng Y. Li B. Dai R. (2019). On the role of synthesized hydroxylated chalcones as dual functional amyloid-β aggregation and ferroptosis inhibitors for potential treatment of Alzheimer’s disease. *Eur. J. Med. Chem.* 166 11–21. 10.1016/j.ejmech.2019.01.039 30684867

[B18] Dixon S. J. Lemberg K. M. Lamprecht M. R. Skouta R. Zaitsev E. M. Gleason C. E. (2012). Ferroptosis: An iron-dependent form of nonapoptotic cell death. *Cell* 149 1060–1072. 10.1016/j.cell.2012.03.042 22632970 PMC3367386

[B19] Fischer R. Maier O. (2015). Interrelation of oxidative stress and inflammation in neurodegenerative disease: Role of TNF. *Oxid. Med. Cell. Longev.* 2015:610813. 10.1155/2015/610813 25834699 PMC4365363

[B20] Guo Q. Wang C. Xue X. Hu B. Bao H. (2021). SOCS1 mediates berberine-induced amelioration of microglial activated states in N9 microglia exposed to β amyloid. *BioMed Res. Int.* 2021:9311855. 10.1155/2021/9311855 34778460 PMC8589517

[B21] Heneka M. T. Carson M. J. El Khoury J. Landreth G. E. Brosseron F. Feinstein D. L. (2015). Neuroinflammation in Alzheimer’s disease. *Lancet Neurol.* 14 388–405. 25792098 10.1016/S1474-4422(15)70016-5 PMC5909703

[B22] Hou W. Xie Y. Song X. Sun X. Lotze M. T. Zeh H. J. III (2016). Autophagy promotes ferroptosis by degradation of ferritin. *Autophagy* 12 1425–1428. 10.1080/15548627.2016.1187366 27245739 PMC4968231

[B23] Jakaria M. Bush A. I. Ayton S. (2021). Ferroptosis as a mechanism of neurodegeneration in Alzheimer’s disease. *J. Neurochem.* 159 804–825. 10.1111/jnc.15519 34553778

[B24] Ji Q. Wang X. Cai J. Du X. Sun H. Zhang N. (2019). MiR-22-3p regulates amyloid β deposit in mice model of Alzheimer’s disease by targeting mitogen-activated protein kinase 14. *Curr. Neurovasc. Res.* 16 473–480. 10.2174/1567202616666191111124516 31713484

[B25] Kheiri G. Dolatshahi M. Rahmani F. Rezaei N. (2018). Role of p38/MAPKs in Alzheimer’s disease: Implications for amyloid beta toxicity targeted therapy. *Rev. Neurosci.* 30 9–30. 10.1515/revneuro-2018-0008 29804103

[B26] Kim S. W. Kim Y. Kim S. E. An J. Y. (2021). Ferroptosis-related genes in neurodevelopment and central nervous system. *Biology (Basel)* 10:35. 10.3390/biology10010035 33419148 PMC7825574

[B27] Lazarov O. Hollands C. (2016). Hippocampal neurogenesis: Learning to remember. *Prog. Neurobiol.* 13 1–18. 10.1016/j.pneurobio.2015.12.006 26855369 PMC4828289

[B28] Le Grand J. N. Chakrama F. Z. Seguin-Py S. Fraichard A. Delage-Mourroux R. Jouvenot M. (2011). GABARAPL1 (GEC1): Original or copycat? *Autophagy* 7 1098–1107. 10.4161/auto.7.10.15904 21597319

[B29] Lueg G. Gross C. C. Lohmann H. Johnen A. Kemmling A. Deppe M. (2015). Clinical relevance of specific T-cell activation in the blood and cerebrospinal fluid of patients with mild Alzheimer’s disease. *Neurobiol. Aging* 36 81–89. 10.1016/j.neurobiolaging.2014.08.008 25277040

[B30] Majerníková N. den Dunnen W. F. A. Dolga A. M. (2021). The potential of Ferroptosis-targeting therapies for Alzheimer’s disease: From mechanism to transcriptomic analysis. *Front. Aging Neurosci.* 13:745046. 10.3389/fnagi.2021.745046 34987375 PMC8721139

[B31] Millot P. San C. Bennana E. Porte B. Vignal N. Hugon J. (2020). STAT3 inhibition protects against neuroinflammation and BACE1 upregulation induced by systemic inflammation. *Immunol. Lett.* 228 129–134. 10.1016/j.imlet.2020.10.004 33096140

[B32] Milovanovic J. Arsenijevic A. Stojanovic B. Kanjevac T. Arsenijevic D. Radosavljevic G. (2020). Interleukin-17 in chronic inflammatory neurological diseases. *Front. Immunol.* 11:947. 10.3389/fimmu.2020.00947 32582147 PMC7283538

[B33] More J. Galusso N. Veloso P. Montecinos L. Finkelstein J. P. Sanchez G. (2018). N-acetylcysteine prevents the spatial memory deficits and the redox-dependent RyR2 decrease displayed by an Alzheimer’s disease rat model. *Front. Aging Neurosci.* 10:399. 10.3389/fnagi.2018.00399 30574085 PMC6291746

[B34] Naughton B. J. Duncan F. J. Murrey D. A. Meadows A. S. Newsom D. E. Stoicea N. (2015). Blood genome-wide transcriptional profiles reflect broad molecular impairments and strong blood–brain links in Alzheimer’s disease. *J. Alzheimers Dis.* 43 93–108. 10.3233/JAD-140606 25079797 PMC5777140

[B35] Olsen I. Singhrao S. K. (2016). Inflammasome involvement in Alzheimer’s disease. *J. Alzheimers Dis.* 54 45–53. 10.3233/JAD-160197 27314526

[B36] Olsson B. Lautner R. Andreasson U. Öhrfelt A. Portelius E. Bjerke M. (2016). CSF and blood biomarkers for the diagnosis of Alzheimer’s disease: A systematic review and meta-analysis. *Lancet Neurol.* 15 673–684. 10.1016/S1474-4422(16)00070-3 27068280

[B37] Perga S. Martire S. Montarolo F. Navone N. D. Calvo A. Fuda G. (2017). A20 in multiple sclerosis and Parkinson’s disease: Clue to a common dysregulation of anti-inflammatory pathways? *Neurotox. Res.* 32 1–7. 10.1007/s12640-017-9724-y 28337659

[B38] Readhead B. Haure-Mirande J. V. Funk C. C. Richards M. A. Shannon P. Haroutunian V. (2018). Multiscale analysis of independent Alzheimer’s cohorts finds disruption of molecular, genetic, and clinical networks by human herpesvirus. *Neuron* 99 64–82.e7. 10.1016/j.neuron.2018.05.023 29937276 PMC6551233

[B39] Reichenbach N. Delekate A. Plescher M. Schmitt F. Krauss S. Blank N. (2019). Inhibition of Stat3-mediated astrogliosis ameliorates pathology in an Alzheimer’s disease model. *EMBO Mol. Med.* 11:e9665. 10.15252/emmm.201809665 30617153 PMC6365929

[B40] Ritchie M. E. Phipson B. Wu D. Hu Y. Law C. W. Shi W. (2015). limma powers differential expression analyses for RNA-sequencing and microarray studies. *Nucleic Acids Res.* 43:e47. 10.1093/nar/gkv007 25605792 PMC4402510

[B41] Simunovic F. Yi M. Wang Y. Macey L. Brown L. T. Krichevsky A. M. (2009). Gene expression profiling of substantia nigra dopamine neurons: Further insights into Parkinson’s disease pathology. *Brain* 132(Pt 7) 1795–1809. 10.1093/brain/awn323 19052140 PMC2724914

[B42] Sood S. Gallagher I. J. Lunnon K. Rullman E. Keohane A. Crossland H. (2015). A novel multi-tissue RNA diagnostic of healthy ageing relates to cognitive health status. *Genome Biol.* 16:185. 10.1186/s13059-015-0750-x 26343147 PMC4561473

[B43] Sutphen C. L. Jasielec M. S. Shah A. R. Macy E. M. Xiong C. Vlassenko A. G. (2015). Longitudinal cerebrospinal fluid biomarker changes in preclinical Alzheimer disease during middle age. *JAMA Neurol.* 72 1029–1042. 10.1001/jamaneurol.2015.1285 26147946 PMC4570860

[B44] Szklarczyk D. Franceschini A. Wyder S. Forslund K. Heller D. Huerta-Cepas J. (2015). STRING v10: Protein–protein interaction networks, integrated over the tree of life. *Nucleic Acids Res.* 43 D447–D452. 10.1093/nar/gku1003 25352553 PMC4383874

[B45] Tang R. Liu H. (2019). Identification of temporal characteristic networks of peripheral blood changes in Alzheimer’s disease based on weighted gene co-expression network analysis. *Front. Aging Neurosci.* 11:83. 10.3389/fnagi.2019.00083 31178714 PMC6537635

[B46] Uddin M. S. Stachowiak A. Mamun A. A. Tzvetkov N. T. Takeda S. Atanasov A. G. (2018). Autophagy and Alzheimer’s disease: From molecular mechanisms to therapeutic implications. *Front. Aging Neurosci.* 10:4. 10.3389/fnagi.2018.00004 29441009 PMC5797541

[B47] Vitalakumar D. Sharma A. Flora S. J. S. (2021). Ferroptosis: A potential therapeutic target for neurodegenerative diseases. *J. Biochem. Mol. Toxicol.* 35 e22830. 10.1002/jbt.22830 34047408

[B48] Wang X. Wang D. Su F. Li C. Chen M. (2022). Immune abnormalities and differential gene expression in the hippocampus and peripheral blood of patients with Alzheimer’s disease. *Ann. Transl. Med.* 10:29. 10.21037/atm-21-4974 35282083 PMC8848377

[B49] Xiao F. J. Zhang D. Wu Y. Jia Q. H. Zhang L. Li Y. X. (2019). miRNA-17-92 protects endothelial cells from erastin-induced ferroptosis through targeting the A20-ACSL4 axis. *Biochem. Biophys. Res. Commun.* 515 448–454. 10.1016/j.bbrc.2019.05.147 31160087

[B50] Xu H. Jia J. (2021). Single-cell RNA sequencing of peripheral blood reveals immune cell signatures in Alzheimer’s disease. *Front. Immunol.* 12:645666. 10.3389/fimmu.2021.645666 34447367 PMC8382575

[B51] Yan H. F. Zou T. Tuo Q. Z. Xu S. Li H. Belaidi A. A. (2021). Ferroptosis: Mechanisms and links with diseases. *Signal Transduct. Target. Ther.* 6:49. 10.1038/s41392-020-00428-9 33536413 PMC7858612

[B52] Yu G. Wang L. G. Han Y. He Q. Y. (2012). clusterProfiler: An R package for comparing biological themes among gene clusters. *Omics* 16 284–287. 10.1089/omi.2011.0118 22455463 PMC3339379

[B53] Yu W. Yu W. Yang Y. Lü Y. (2021). Exploring the key genes and identification of potential diagnosis biomarkers in Alzheimer’s disease using bioinformatics analysis. *Front. Aging Neurosci.* 13:602781. 10.3389/fnagi.2021.602781 34194312 PMC8236887

[B54] Zhang G. Zhang Y. Shen Y. Wang Y. Zhao M. Sun L. (2021). The potential role of Ferroptosis in Alzheimer’s disease. *J. Alzheimers Dis.* 80 907–925. 10.3233/JAD-201369 33646161

[B55] Zhang S. Zhang Z. Sandhu G. Ma X. Yang X. Geiger J. D. (2007). Evidence of oxidative stress-induced BNIP3 expression in amyloid beta neurotoxicity. *Brain Res.* 1138 221–230. 10.1016/j.brainres.2006.12.086 17274962

[B56] Zhou B. Liu J. Kang R. Klionsky D. J. Kroemer G. Tang D. (2020). Ferroptosis is a type of autophagy-dependent cell death. *Semin. Cancer Biol.* 66 89–100. 10.1016/j.semcancer.2019.03.002 30880243

[B57] Zhou Y. Zhou B. Pache L. Chang M. Khodabakhshi A. H. Tanaseichuk O. (2019). Metascape provides a biologist-oriented resource for the analysis of systems-level datasets. *Nat. Commun.* 10:1523. 10.1038/s41467-019-09234-6 30944313 PMC6447622

