# Supplementary material for: Exploring the key ferroptosis-related gene in the peripheral blood of patients with Alzheimer’s disease and its clinical significance
Source: Front Aging Neurosci. 2022 Sep 1;14:970796. doi: 10.3389/fnagi.2022.970796 (PMC9475071; doi:10.3389/fnagi.2022.970796)
Supplement: Supplementary file 1 [file Data_Sheet_1.zip › Supplementary Figures.docx]

# Supplementary Figures


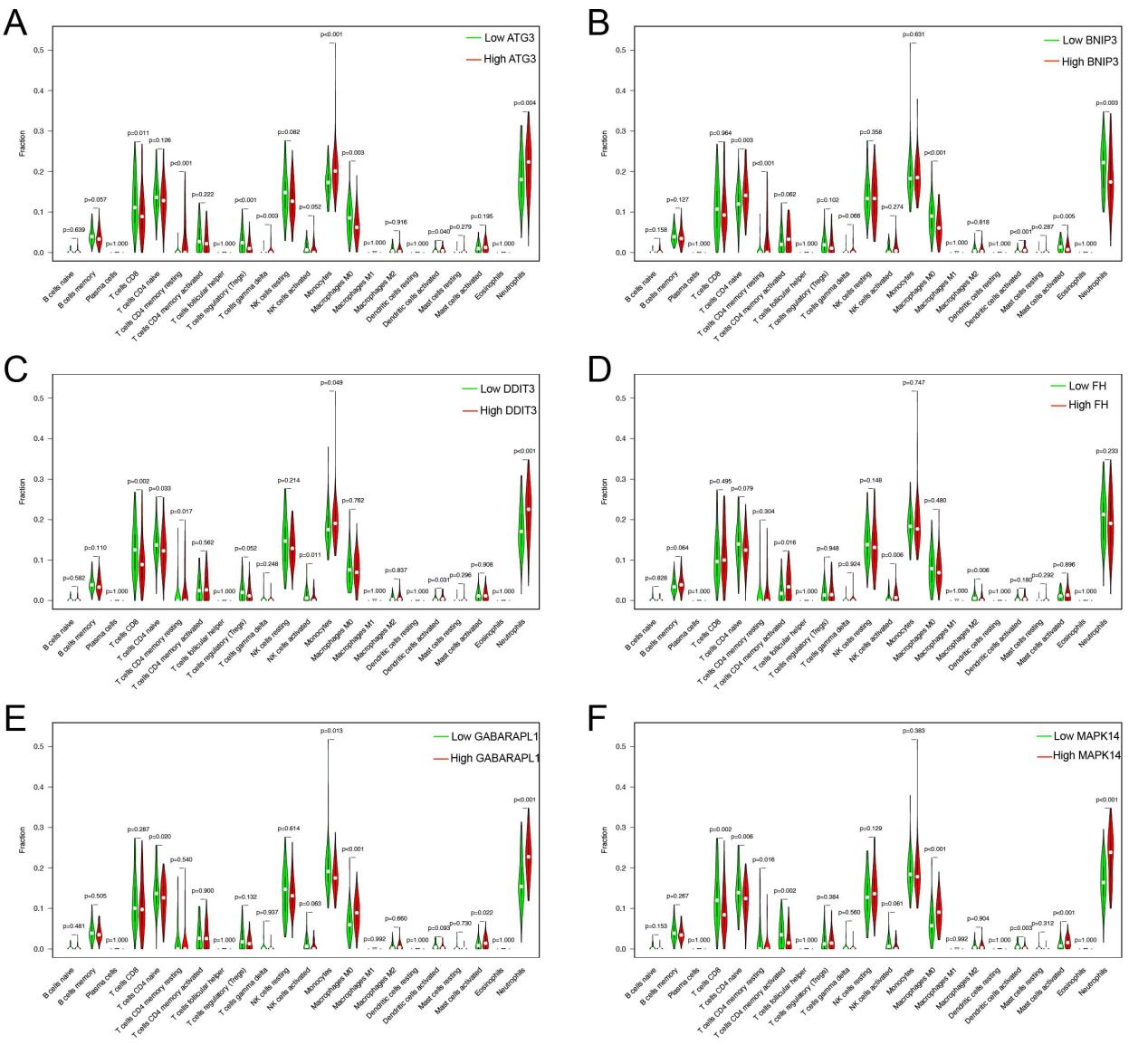


# Supplementary Figure **1**. Proportions of 22 types of immune cells in AD samples with differential expression of ferroptosis-related genes. (A) ATG3; (B) BNIP3; (C) DDIT3; (D) FH; (E) GABARAPL1; (F) MAPK14.


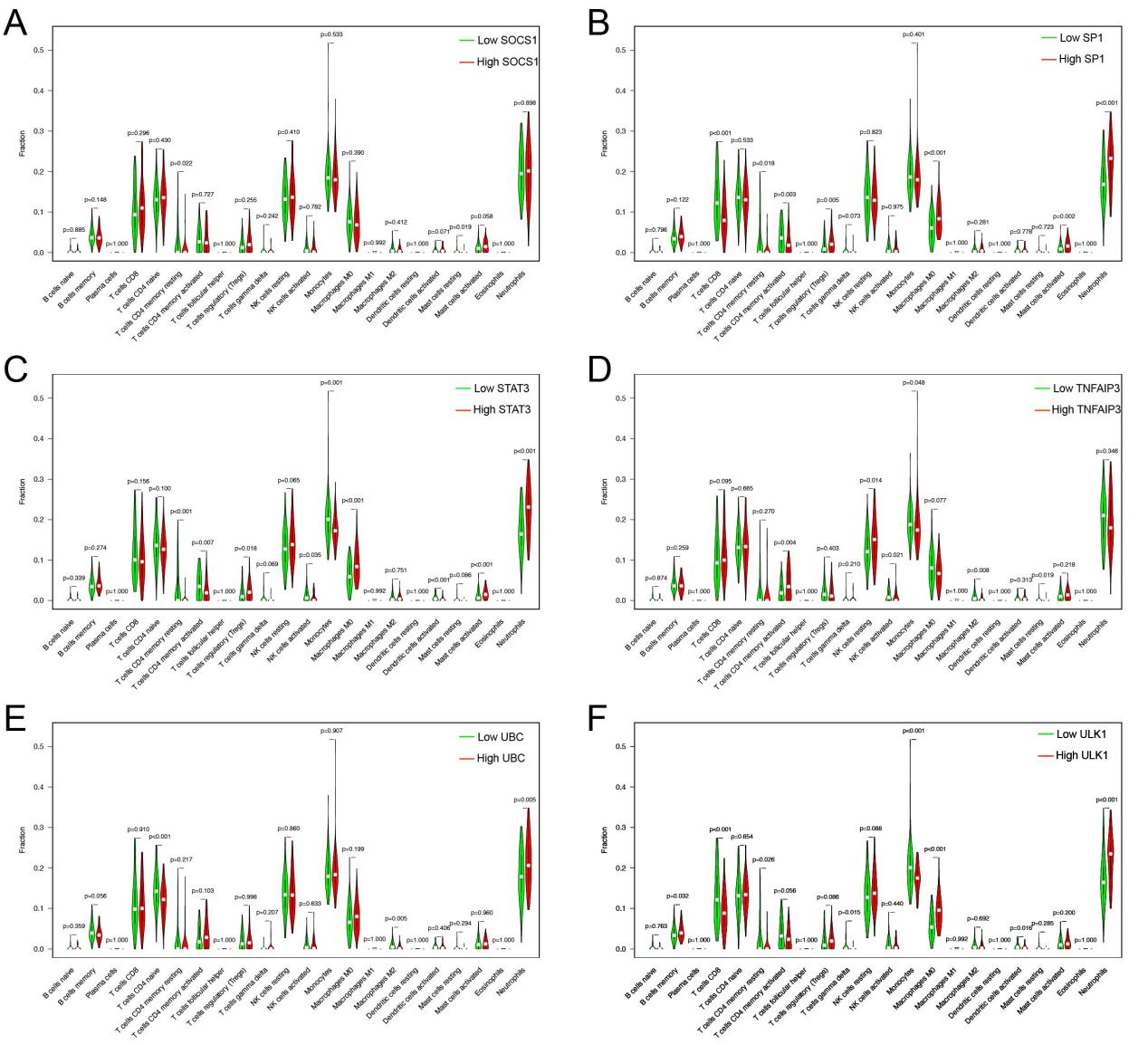


# Supplementary Figure 2. Proportions of 22 types of immune cells in AD samples with differential expression of ferroptosis-related genes. (A) SOCS1; (B) SP1; (C) STAT3; (D) TNFAIP3; (E) UBC; (F) ULK1.
